# Interpenetrating network hydrogels for studying the role of matrix viscoelasticity in 3D osteocyte morphogenesis†

**DOI:** 10.1039/d3bm01781h

**Published:** 2024-01-10

**Authors:** Margherita Bernero, Doris Zauchner, Ralph Müller, Xiao-Hua Qin

**Affiliations:** a Institute for Biomechanics, ETH Zürich Switzerland qinx@ethz.ch

## Abstract

During bone formation, osteoblasts are embedded in a collagen-rich osteoid tissue and differentiate into an extensive 3D osteocyte network throughout the mineralizing matrix. However, how these cells dynamically remodel the matrix and undergo 3D morphogenesis remains poorly understood. Although previous reports investigated the impact of matrix stiffness in osteocyte morphogenesis, the role of matrix viscoelasticity is often overlooked. Here, we report a viscoelastic alginate–collagen interpenetrating network (IPN) hydrogel for 3D culture of murine osteocyte-like IDG-SW3 cells. The IPN hydrogels consist of an ionically crosslinked alginate network to tune stress relaxation as well as a permissive collagen network to promote cell adhesion and matrix remodeling. Two IPN hydrogels were developed with comparable stiffnesses (4.4–4.7 kPa) but varying stress relaxation times (*t*_1/2_, 1.5 s and 14.4 s). IDG-SW3 cells were pre-differentiated in 2D under osteogenic conditions for 14 days to drive osteoblast-to-osteocyte transition. Cellular mechanosensitivity to fluid shear stress (2 Pa) was confirmed by live-cell calcium imaging. After embedding in the IPN hydrogels, cells remained highly viable following 7 days of 3D culture. After 24 h, osteocytes in the fast-relaxing hydrogels showed the largest cell area and long dendritic processes. However, a significantly larger increase of some osteogenic markers (ALP, Dmp1, hydroxyapatite) as well as intercellular connections *via* gap junctions were observed in slow-relaxing hydrogels on day 14. Our results imply that fast-relaxing IPN hydrogels promote early cell spreading, whereas slow relaxation favors osteogenic differentiation. These findings may advance the development of 3D *in vivo*-like osteocyte models to better understand bone mechanobiology.

## Abstract 2

3D osteocyte cultures reveal that fast stress-relaxing hydrogels enhance early osteocyte morphogenesis, while slow-relaxing hydrogels favor osteogenic differentiation after 14 days, highlighting their mechanosensitivity to matrix mechanics.

## Graphical abstract

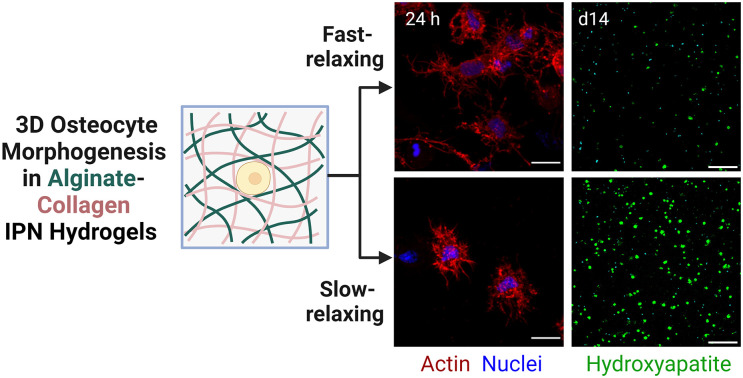

## Introduction

Osteocytes are the key mechanosensors in bone. During bone development, they differentiate from osteoblasts and are first embedded in a collagen-rich osteoid tissue which is mineralized thereafter. Within the lacuno-canalicular system, an extensive osteocyte network forms that can sense load applied to the bone and orchestrate bone remodeling in accordance with it.^1^ However, a detailed understanding of osteocyte morphogenesis throughout this process is lacking. Developing *in vitro* osteocyte models thus has the potential to enrich our fundamental understanding of their mechanobiology in health and disease. To facilitate *in vitro* research on osteocytes, several murine osteocytic cell lines have been established.^2–5^ Amongst them, the IDG-SW3 cell line developed by Woo *et al.* is able to replicate osteoblast-to-osteocyte transition within 28 days and allows direct tracking of the differentiation process *via* the expression of a fluorescent reporter.^6^ This cell line has been a valuable tool to study osteocyte maturation as well as cellular interaction with the surrounding matrix *in vitro*.^7–9^

In the field of mechanobiology, which explores the effect of physical cues on cellular behavior, studying how matrix mechanics influence cell fates has raised increasing interest. Amongst these mechanical properties, matrix stress relaxation was shown to regulate various cellular processes such as cell spreading, proliferation and differentiation in mesenchymal stem cells.^10^ Specifically for osteocytic development, *in vitro* studies have focused on the effect of matrix stiffness, which changes drastically over time as the mineralization of the organic osteoid matrix proceeds. Lower stiffnesses promoted osteogenic differentiation of MC3T3 cells in 3D gelatin hydrogels (0.58 *vs.* 1.47 kPa) and increased mineral deposition for primary osteoprogenitor cells in gelatin methacryloyl (GelMA) gels (6.3 *vs.* 16.3 and 40.2 kPa).^11,12^ However, the viscoelastic nature of native osteoid tissue^13^ is often overlooked and thus, the effect of varying matrix stress relaxation on osteocyte differentiation has not been investigated to date.

**Fig. 1:**
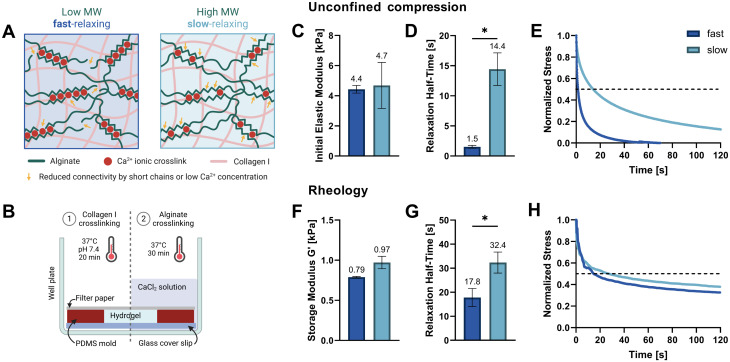
Hydrogel tuning, casting, and mechanical characterization. (A) Tuning stress relaxation properties independently of initial elastic modulus. By reducing the molecular weight (*M*_w_) of alginate polymers, connectivity decreases, and stress relaxation speeds are increased. The connectivity in higher-*M*_w_ gels can be reduced by lowering the Ca^2+^ crosslinker concentration to match the lower-*M*_w_ alginate stiffness. The interpenetrating collagen network which is inherently viscoelastic also contributes to the total stress relaxation speed. (B) Schematic of setup and procedure used for alginate–collagen I gel casting and cell embedding. (C–E) Unconfined compression testing on a Texture Analyser up to a strain of 15% was used to determine the initial elastic moduli (C) and compressive stress relaxation half-times (D) of swollen hydrogels. (E) Means of three normalized stress measurements at a constant strain of 15% are shown. The dashed line marks a decay to half of the initial stress and intersections denote the relaxation half-time. (F–H) Rheological amplitude sweeps were used to determine the storage moduli (F) and a shear stress relaxation was measured at constant 10% shear strain (G). (H) Means of two or three normalized shear stress measurements at constant strain are shown. The raw data was filtered using a median and a low pass filter. (C, D, F and G) Data is presented as mean ± SD, *n* = 3 (except rheology of fast with *n* = 2). Welch's *t* tests were performed to compare elastic moduli, storage moduli and relaxation half-times. *p = 0.05 to 0.01.

In this work, we study how osteocyte-like IDG-SW3 cells respond to different stress relaxation speeds of their surrounding matrix. We hypothesized that a faster relaxing extracellular matrix (ECM) would facilitate cell spreading through matrix reorganization and that this might affect osteocytic differentiation. Thus, we developed fast- and slow-relaxing alginate–collagen interpenetrating network (IPN) hydrogels for 3D osteogenic culture. Alginate is a natural polysaccharide composed of β-d-mannuronic acid (M) and α-l-guluronic acid (G) that forms ionically crosslinked hydrogels in the presence of divalent cations such as Ca^2+^.^14^ The fact that these ionic crosslinks can be broken and reformed when experiencing strain, renders them stress relaxing.^15^ These stress relaxation properties can readily be tuned by lowering the molecular weight (*M*_w_) of the alginate polymer, which decreases connectivity and thereby increases stress relaxation speed. Adjusting the stiffness is possible independently of the alginate *M*_w_ by increasing or decreasing the Ca^2+^ crosslinker concentration (Fig. 1A).^16^

Collagen type I, the main component of osteoid, has been shown to promote IDG-SW3 osteogenic differentiation and matrix mineralization *in vitro*. Yang *et al.* have investigated the incorporation of collagen I into a GelMA–hyaluronic acid methacrylate (HAMA) bioink for 3D bioprinting of osteocyte-laden constructs. Increased functionality, dendrite number and length of 3D embedded IDG-SW3 were observed with increasing concentrations of collagen I in the bioink.^9^ Therefore, collagen I was included in our IPN hydrogel compositions that combine the stress relaxation properties of alginate with the permissiveness of collagen.

We established fast-relaxing (*t*_1/2_, 1.5 s) and slow-relaxing (*t*_1/2_, 14.4 s) IPN hydrogels as an *in vitro* platform to address the lack of osteocyte mechanobiology studies investigating matrix viscoelasticity. For this 3D culture system, pre-differentiated IDG-SW3 cells were characterized and used as an osteocyte model. For the first time, Ca^2+^ imaging was reported in this cell line as a tool for real-time visualization of cellular mechanotransduction. We then characterized the cell and tissue development under osteogenic conditions to identify the effects of different matrix stress relaxation speeds. This study provides new insights into osteocyte morphogenesis and differentiation in viscoelastic 3D microenvironments.

## Results and discussion

### Hydrogel casting and mechanical properties

Two soft IPN hydrogel compositions were developed with 0.3% (w/v) alginate and 0.3% (w/v) collagen I. Very low viscosity alginate (VLVG, *M*_w_ < 75 kDa) was used to produce fast-relaxing gels (fast) while medium viscosity alginate (MVG, *M*_w_ > 200 kDa) was used for slow-relaxing gels (slow). A sequential hydrogel crosslinking protocol was adapted from Gillette *et al.*,^17^ starting with the thermal gelation of collagen, followed by alginate crosslinking with a CaCl_2_ solution which provided the divalent Ca^2+^ cations required for ionic crosslinking (Fig. 1B). To exclude the effect of stiffness variation due to different alginate molecular weights and match the gel stiffnesses of both compositions, a range of CaCl_2_ concentrations was tested and the resulting mechanical properties screened. Finally, 20 mM CaCl_2_ were selected for the fast-relaxing gels while slow-relaxing gels were crosslinked with 5 mM CaCl_2_. Previous studies with mesenchymal stem cells have employed the same stiffness tuning approach and shown that differences in CaCl_2_ concentration do not affect cell viability and functionality in 3D cultures *in vitro*.^10,18^

The initial elastic moduli of acellular hydrogels were computed by unconfined compression tests and resulted in a range of 4.4–4.7 kPa for the selected conditions (Fig. 1C and Table 1). Stress relaxation to compressive strain was also evaluated and a significant difference in relaxation half-time *t*_1/2_ was found between the fast- and slow-relaxing gels (1.5 s and 14.4 s, respectively, Fig. 1D). It should however be noted that overall, the stress relaxation half-times are on a smaller magnitude range than similar studies performed with mesenchymal stem cells (∼1 min to ∼1 h by Chaudhuri *et al.* and ∼30 s to ∼13 min by Darnell *et al.*).^10,18^ Thus, the slow-relaxing composition still exhibits substantial stress relaxation, which is also shown in Fig. 1E. This can mainly be attributed to the inclusion of collagen, a viscoelastic material itself that further increases the IPN stress relaxation speed.

**Table 1:** Mechanical characterization of fast- and slow-relaxing formulations

	Unconfined compression		Rheology	
	Init. elastic modulus [kPa]	Relaxation time *t*_1/2_ [s]	Storage modulus *G*′ [kPa]	Relaxation time *t*_1/2_ [s]
Fast	4.429 ± 0.253	1.5 ± 0.2	0.789 ± 0.009	17.8 ± 3.8
Slow	4.684 ± 1.526	14.4 ± 2.7	0.972 ± 0.077	32.35 ± 4.4

The second method used to mechanically characterize the casted hydrogels was *in situ* rheology. Using amplitude sweeps to determine the linear viscoelastic range and storage moduli as a measure of stiffness, the relative stiffnesses found in compression tests could be confirmed (0.79–0.97 kPa, Fig. 1F and ESI Fig. S1†). The difference in shear stress relaxation was also significant, although less pronounced with stress relaxation half-times *t*_1/2_ of 17.8 s for fast-relaxing gels and 32.4 s for the slow-relaxing condition (Fig. 1G). A difficulty for the analysis of the rheological stress relaxation tests was the relatively large noise at the beginning of the measurements caused by a high sampling rate to capture the initial relaxation kinetics. Such noise has also been reported previously^19^ but filtering it out prior to evaluation allowed the generation of relatively smooth stress relaxation curves (Fig. 1H).

### Characterization of 2D pre-differentiated IDG-SW3

With the goal of establishing an osteocyte-like 3D culture within the newly developed gels, we used the murine bone cells IDG-SW3 ^6^ and first pre-differentiated them in 2D for 14 days. This cell line is expanded in an osteoblast-like state when interferon γ (INF-γ) is present, which regulates the expression of a thermolabile SV40 T antigen and sustains a proliferative state at 33 °C. Differentiation along the osteogenic lineage towards osteocytes is induced upon removal of INF-γ and culture temperature increase to 37 °C. To track the early osteocyte marker dentin matrix acidic phosphoprotein 1 (Dmp1), the cell line also includes a green fluorescent protein marker driven by a Dmp1 promoter (Dmp1-GFP) that allows to fluorescently label cells that have transitioned into an early osteocyte stage.

**Fig. 2:**
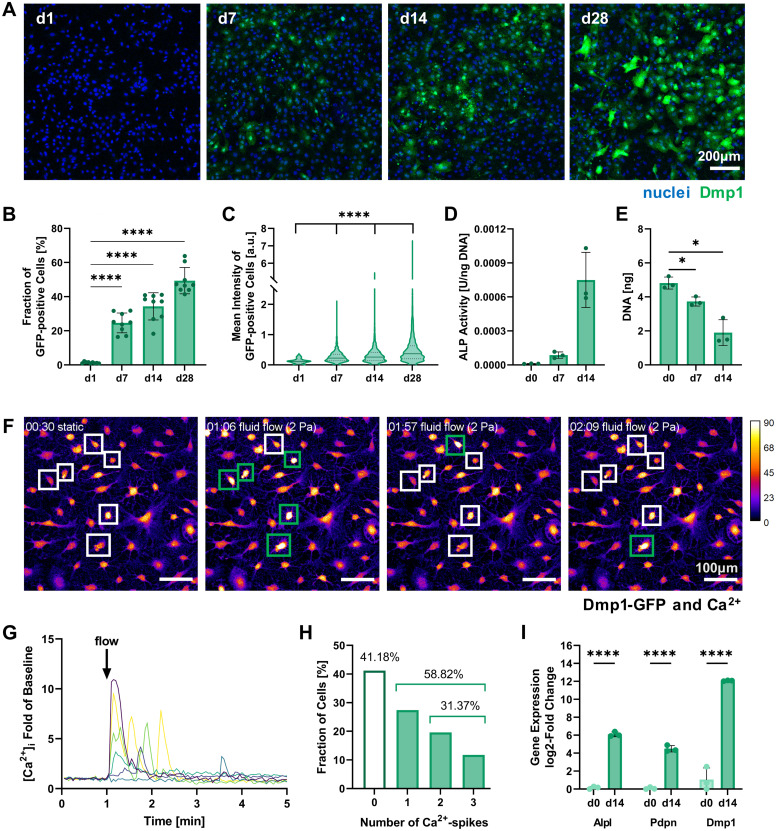
Characterization of 2D pre-differentiating IDG-SW3 cells. (A) Representative fluorescence microscopy maximum intensity projections on days 1, 7, 14 and 28 of 2D culture under osteogenic conditions used for quantitative analysis of Dmp1-GFP expression. (B and C) The fraction of Dmp1-GFP-positive cells at all time points and each fluorescent cell's mean intensity were quantified using the CellProfiler software. (D) ALP activity quantified by a colorimetric assay and normalized to the DNA content. Welch's one-way ANOVA showed a significant difference among means (*p* = 0.05 to 0.01), however, pairwise comparisons were non-significant. (E) DNA content of initially 0.5 × 10^6^ seeded cells quantified by Quant-iT PicoGreen dsDNA assay. (F–H) Mechanoresponse of pre-differentiated IDG-SW3 visualized by live cell Ca^2+^ imaging under fluid flow in 2D on day 14. Cells were stained with Fluo-4 to emit a Ca^2+^-concentration dependent fluorescent signal when a fluid shear stress of 2 Pa was applied. See ESI Fig. S2† for a schematic depiction of the experimental setup. (F) Maximum intensity projections of the baseline under static conditions and at three time points during fluid flow application are shown visualizing fluorescence intensity (Dmp1-GFP and Ca^2+^ signal overlapping). Selected responsive cells are highlighted and green boxes denote a Ca^2+^ spike. See also ESI Video S1.† (G) Normalized intensity signals of selected responsive cells are shown over time. Each line represents an individual cell and most cells respond immediately after fluid flow onset at 1 minute. (H) Mean intensities of all cells were analyzed individually over time and the number of spikes was recorded. (I) Gene expression of markers for osteogenic differentiation (Alpl: osteoblast, Pdpn and Dmp1: early osteocyte, B2m as reference gene) was quantified by RT-qPCR before and after the two-week pre-differentiation period. (B, D, E and I) Data is presented as mean ± SD, *n* = 3. Welch's one-way ANOVA with Dunnett's T3 multiple comparisons test or two-way ANOVA with Šídák's multiple comparisons test were performed. (C) Data is shown with median (solid line) and quartiles (dotted line), *n* = 75–2631. A Kruskal–Wallis test followed by Dunn's multiple comparisons test was performed. *p = 0.05 to 0.01, ****p ≤ 0.0001.

Multiple assays including this marker were used to assess the successful pre-differentiation of IDG-SW3 after 14 days in 2D culture. Quantification of Dmp1-GFP-expressing cells by confocal fluorescence microscopy showed that around 34% of cells had reached an osteocytic phenotype by day 14 and nearly 50% of all cells were GPF-positive by day 28 (Fig. 2A and B). These results fail to reach the levels reported in the original IDG-SW3 publication^6^ and could indicate a slower differentiation progress in our culture. Apart from the continuous increase in the fraction of Dmp1-expressing cells, the fluorescent intensity of GFP-positive cells also significantly increased until day 28 (Fig. 2C). This implies that gradually more and more cells differentiate, reach an early osteocyte stage, and continue to mature during the 28 days of osteogenic culture. Thus, a similar continued development can also be expected for 14-days pre-differentiated IDG-SW3 used for 3D cell embedding.

Dmp1 gene expression during culture in osteogenic conditions was subsequently confirmed using real-time quantitative PCR (RT-qPCR). After 14 days, Dmp1 expression showed a significant increase with a log_2_-fold change above 10 compared to the expansion cells (Fig. 2I). Additionally, the osteoblast differentiation marker alkaline phosphatase (Alpl) and early osteocyte marker podoplanin (Pdpn, also known as E11) were analyzed by RT-qPCR. Both genes showed significant upregulation after differentiation was induced.

Alkaline phosphatase enzyme (ALP) activity can also be measured on the protein level and has been used to characterize IDG-SW3 during osteoblast-to-osteocyte transition when the cell line was first reported, peaking on day 14.^6^ ALP activity was evaluated for our 2D cell cultures up to day 14 using a colorimetric assay and showed a strong increasing trend (Fig. 2D). DNA contents of the samples were quantified for ALP assay normalization and revealed a significant decrease (Fig. 2E). We assume that some cells are lost during the differentiation process, either by failing to attach properly to the culture flask surface or by apoptosis.

To further verify the mechanosensitivity of the pre-differentiated IDG-SW3 cells, a protocol for live cell calcium (Ca^2+^) imaging under fluid flow stimulation was developed. Osteocytes have been shown to be especially responsive to fluid flow. Such fluid shear stresses can induce the opening of stretch-activated ion channels in the cell membrane, causing a Ca^2+^ influx from the extracellular fluid and the endoplasmatic reticulum that initiates an intracellular mechanotransduction cascade.^20–22^ Visualizing changes in Ca^2+^ concentration therefore allows the tracking of mechanosensation in real-time. Applying a shear stress of 2 Pa in a commercial ibidi μ-Slide, we demonstrate the successful Ca^2+^ visualization in pre-differentiated IDG-SW3 using the Ca^2+^-indicator Fluo-4 (Fig. 2F). The inclusion of Pluronic F-127, a dispersing agent increasing Fluo-4 AM solubility and uptake into the cells, was found to be crucial to ensure sufficient dye loading for Ca^2+^ imaging. Despite the emission spectrum overlaying with Dmp1-GFP, it was possible to detect Ca^2+^ spikes upon fluid flow stimulation (Fig. 2G and ESI Video S1†). Almost 60% of the analyzed IDG-SW3 responded to the 2 Pa shear stress and most of them spiked immediately after fluid flow onset. Additionally, more than half of the responsive cells were able to produce multiple spikes over the course of 4 minutes (Fig. 2H). Although calcium spikes had previously been recorded in response to fluid shear stress for MLO-Y4 cells, another murine osteocyte-like cell line,^22^ this is the first account of live cell calcium imaging with IDG-SW3 cells. Having confirmed the mechanosensitivity of 14-days pre-differentiated IDG-SW3 will allow us to extend our studies of osteocyte mechanobiology to 3D settings *in vitro*.

### 3D cell culture establishment in IPN hydrogels

**Fig. 3:**
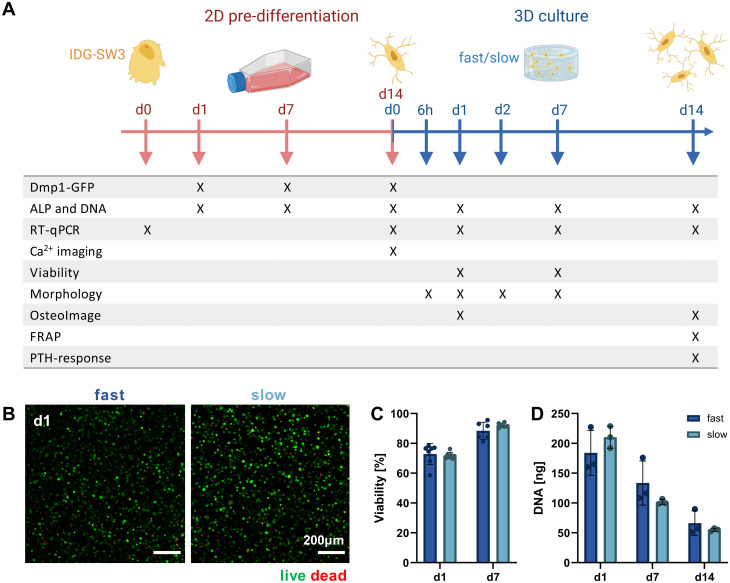
Validating 3D cell cultures of pre-differentiated IDG-SW3 cells in alginate–collagen hydrogels. (A) Timeline of experiments conducted during 2D pre-differentiation and after 3D embedding of IDG-SW3 cells cultured under osteogenic conditions. (B) Representative maximum intensity projections of cells in fast- and slow-relaxing hydrogels stained with calcein AM (live) and ethidium homodimer 1 (dead) on day 1. (C) Cell viability on days 1 and 7 was quantified from the live–dead stained samples. (D) DNA content of 3D samples over the course of 14 days was quantified by Quant-iT PicoGreen dsDNA assay. (C and D) Data is presented as mean ± SD, (C) *n* = 2, (D) *n* = 3. Two-way ANOVA with Šídák's multiple comparisons test was performed.

Mimicking the osteocyte embedding process in osteoid tissue *in vivo*,^23^ we embedded the pre-differentiated IDG-SW3 cells in the fast- and slow-relaxing IPN hydrogels to evaluate cell morphogenesis and maturation in response to varying matrix stress relaxation speeds over the course of another 14 days. A timeline of the conducted experiments and investigated time points over the total 28 days including the pre-differentiation period can be seen in Fig. 3A.

Pre-differentiated IDG-SW3 were directly suspended in the hydrogel precursor solution at a concentration of 4.5 × 10^6^ cells per mL and embedded in the IPN hydrogels analogously to acellular gel casting (Fig. 1B). To validate the system for 3D culture compatibility, the cell viability was quantified over 7 days after embedding. Viabilities did not differ significantly between fast- and slow-relaxing hydrogels but were overall lower on day 1 with around 75% (Fig. 3B and C). However, viability increases until day 7 where it reaches around 90% for both groups, indicating that the IPN hydrogels support IDG-SW3 culture *in vitro*.

Further insights can also be gained from the DNA quantification that was performed in combination with the ALP assay for its normalization. Both conditions showed a comparable decrease from day 1 to days 7 and 14 (Fig. 3D). This may partly be explained by the loss of cells migrating out of the gels into the culture medium, as cells had been observed growing on the culture well bottoms. Osteogenic differentiation followed by apoptosis may be a second factor causing a reduction in DNA, which is in line with DNA decreases during 2D pre-differentiation and was also noted in previous studies working with mesenchymal stem cells under osteogenic conditions in 3D.^24,25^ However, quantification of pro-apoptotic (Bax) and anti-apoptotic (Bcl-2) marker gene expression indicates that compared to 2D pre-differentiation, there is still a stronger apoptotic stress on cells in osteogenic 3D culture that decreases until day 14. This may be related to the cellular stress during embedding (ESI Fig. S3†).

### Analysis of 3D cellular morphology

**Fig. 4:**
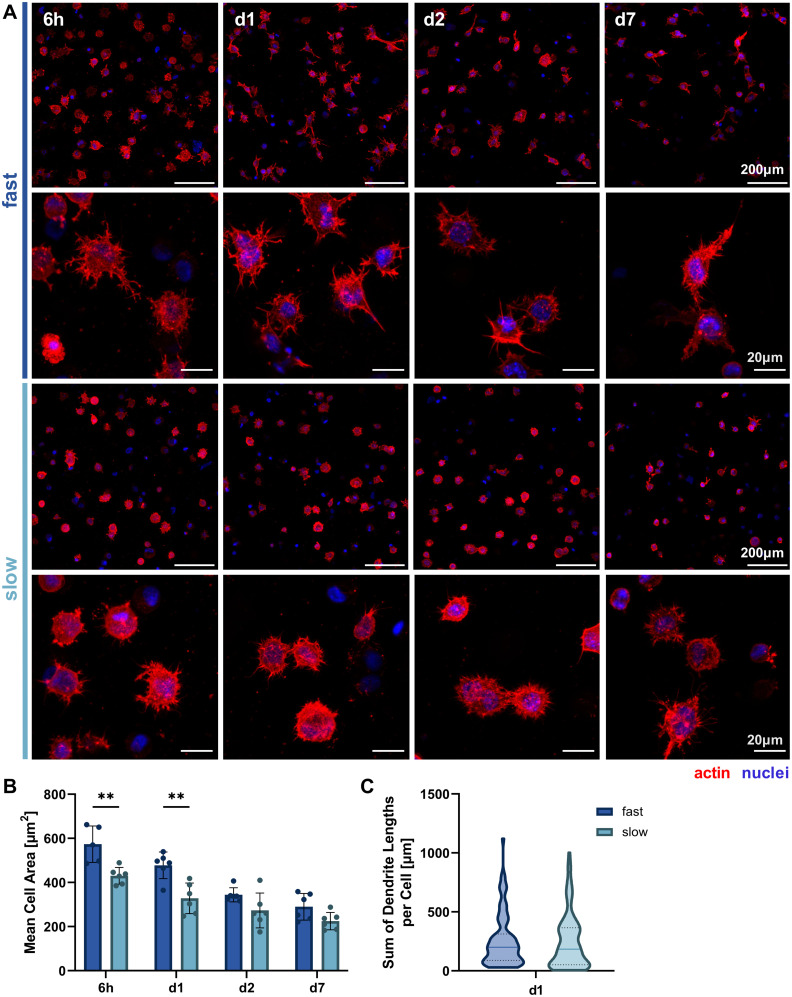
Morphological analysis of 3D cultures in hydrogels of varying stress relaxation speeds. (A) Representative fluorescence microscopy maximum intensity projections of cells in fast- and slow-relaxing conditions stained with Hoechst (nuclei) and phalloidin-TRITC (actin) after 6 h, 24 h (d1), 48 h (d2) and 7 days. (B) Mean cell area over the time course quantified based on the actin signal. Data is presented as mean ± SD, *n* = 3. Two-way ANOVA with Šídák's multiple comparisons test for differences between stress relaxation speeds was performed. **p = 0.01 to 0.001. (C) Total dendrite length quantified using the IMARIS automated dendrite tracking tool for cells embedded on day 1. Data is shown with median (solid line) and quartiles (dotted line), *n* = 87–106. A Mann Whitney test was performed and no significant difference between medians was found.

Since we hypothesized that faster stress relaxation would allow the embedded cells to spread more easily by matrix reorganization in addition to degradation-dependent spreading, we analyzed cellular morphology by actin-nuclei-stained samples at multiple timepoints throughout the first week of 3D culture (6 h, day 1, day 2, day 7, Fig. 4A). This allowed the qualitative and quantitative evaluation of early osteocyte morphogenesis in our fast- and slow-relaxing IPN hydrogels. The most promising morphologies with dendrites extending from the cell body and visible cell–cell-connections were found in cells embedded in the fast-relaxing gels between 6 h and 24 h (day 1). This early and active spreading is also visible in the cell area quantification based on the actin signal (Fig. 4B). The mean cell area is significantly larger with fast-relaxing IPN hydrogels until day 1. The trend persists until day 7, although cell area generally decreases for both conditions and no significant difference can be detected by day 2. This reduction in cell area can also be an indicator of osteoblast-to-osteocyte transition.^20^ Despite the lower mean cell area in slow-relaxing gels, a stellate morphology with a multitude of thin dendrites can be observed that more closely resembles the typical osteocyte morphology *in vivo* with an ellipsoid cell body and long dendrites.

Finally, the sum of dendrite lengths was also quantified from the actin-nuclei *z*-stacks on day 1 using an automated dendrite tracking tool. The longest dendrite lengths were measured in the fast-relaxing composition which also showed a slightly higher median length (Fig. 4C). The difference, however, was not statistically significant and future more highly resolved studies of individual dendrite lengths might be of interest to capture the visible difference in the microscopy images.

### Molecular biomarkers during 3D osteocytic differentiation

**Fig. 5:**
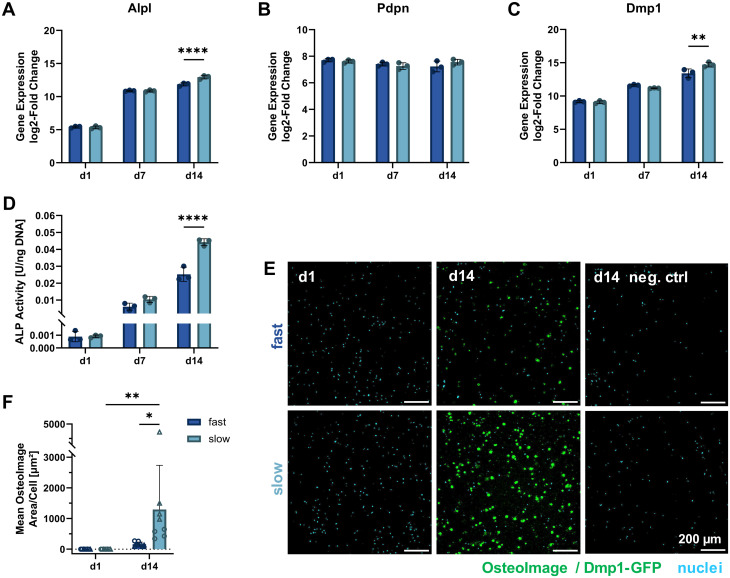
Molecular cell markers of differentiation and matrix mineralization under osteogenic 3D culture. (A–C) Gene expression quantification of osteogenic markers (Alpl: osteoblast, Pdpn and Dmp1: early osteocyte, B2m as reference gene) by RT-qPCR for 3D cultures over 14 days. The fold change is shown relative to a single reference point for all genes. (D) ALP activity in 3D samples quantified on days 1, 7 and 14 of osteogenic culture by a colorimetric assay and normalized to the DNA content (see Fig. 3D). (E) To visualize internal matrix mineralization over the course of 14 days, maximum intensity projections of 20 μm sections stained with OsteoImage reagent and Hoechst for hydroxyapatite and nuclei, respectively, are shown. A negative control without OsteoImage staining was included to verify a negligible signal contribution by Dmp1-GFP. (F) The mean OsteoImage signal area normalized by the number of cells was quantified form the fluorescence images. Data points with the same symbol (circles, triangles) represent different positions within the same sample. (A–D and F) Data is presented as mean ± SD, (A–D) *n* = 3, (F) *n* = 2. Two-way ANOVA with Šídák's multiple comparisons test was performed. *p = 0.05 to 0.01, **p = 0.01 to 0.001, ****p ≤ 0.0001.

To further study the differentiation of IDG-SW3 cells during 3D culture, several osteogenic marker gene transcripts were quantified by RT-qPCR on days 1, 7 and 14 (corresponding to days 15, 21 and 28 of total osteogenic culture). A steady increase in Alpl expression indicated continued osteoblastic differentiation with a significantly higher level in slow-relaxing gels on day 14 (Fig. 5A). This was unexpected, compared to previous reports of IDG-SW3 differentiation both in 2D and 3D that had shown a peak around day 14.^6,7^ Also the two early osteocyte markers Pdpn and Dmp1 were investigated in 3D by RT-qPCR. Pdpn remains at a constant and high expression level (Fig. 5B), which is in line with the cell line creators’ observation of sustained elevated levels after peaking around day 7.^6^ Dmp1 expression further increased during osteogenic 3D culture and after 14 days was significantly higher in slow-relaxing gels suggesting that decreased relaxation speeds may promote differentiation over time (Fig. 5C).

Furthermore, ALP enzyme activity was assayed on days 1, 7 and 14 in 3D culture. In agreement with the RT-qPCR data, ALP activity also increased on the protein level and was significantly higher in the slow-relaxing condition on day 14 (Fig. 5D). This disagreement with reports in literature might be due to differentiation proceeding more slowly in our cultures, which had already been indicated by the Dmp1-GFP-positive cell fraction during osteogenic culture in 2D. Additional stresses may be put on the cells during dissociation after pre-differentiation, the cell embedding procedure and adaptation to a 3D culture environment.

As the embedded cells mature, we also expect changes in the surrounding matrix due to proteolytic remodeling, secretion of matrix proteins and induced mineral deposition. Thus, matrix mineralization was investigated using OsteoImage staining for hydroxyapatite. Despite a spectral overlap with the Dmp1-GFP signal, OsteoImage was detectable in mineralized nodules on day 14 (Fig. 5E). At the selected image contrast settings, barely any Dmp1-GFP signal can be seen in the negative control without OsteoImage staining. The signal from the fluorescence microscopy images was quantified and normalized to the cell number (Fig. 5F). A significant difference was found for slow-relaxing gels on day 14 both compared to the baseline sample on day 1 and compared to the fast-relaxing gels on day 14. This enhanced mineralization can also be observed in histological sections stained with Alizarin Red S (ESI Fig. S4†) and confirms our findings of increased differentiation markers in slow-relaxing hydrogels at the end timepoint day 14. To verify whether the cell-induced matrix remodeling and mineralization had an effect on bulk gel mechanical properties, unconfined compression tests were performed on days 1, 7 and 14. These did not reveal any evident changes of elastic modulus over time and showed that the hydrogels retained their initial stress relaxation properties (ESI Fig. S5†). Nevertheless, the local mechanical environment may undergo changes undetectable by bulk measurements. Future investigations into the dynamics of cellular matrix remodeling are therefore warranted. Newly deposited matrix greatly influences how cells sense their local environment, which could for example be visualized by nascent protein labelling.^26^

### Functional analysis of 3D osteocyte cultures

**Fig. 6:**
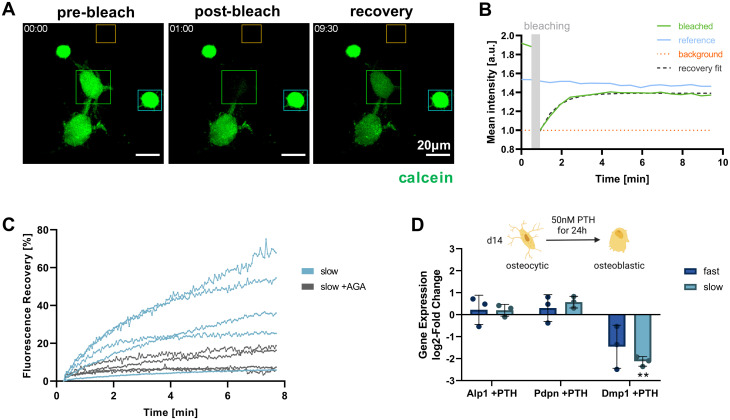
Functional connectivity and response of IDG-SW3 cells in 3D culture. (A–C) Fluorescence recovery after photobleaching (FRAP) experiment to validate cell–cell interconnectivity *via* dye transfer over gap junctions. (A) Maximum intensity projections of pre-bleach, post-bleach and recovery timepoints during a FRAP experiment. Cells on day 14 in 3D culture were stained with calcein AM as transferrable dye. Target cells were subsequently bleached with 488 nm laser irradiation and recovery was monitored by time-lapsed live cell imaging. (B) Fluorescence intensity quantification of the regions of interest marked with the corresponding color in panel (A) to visualize the experimental procedure. (C) FRAP of single cells in slow-relaxing gels, each line representing a cell that is connected to at least one other cell. 100 mM 18α-glycyrrhetinic acid (AGA), a gap junction inhibitor, was used as a negative control for recovery *via* dye transfer. Fluorescence recovery over time was quantified from the target cell intensity relative to the post-bleach intensity and normalized by the pre-bleach intensity. (D) Response of 3D-embedded cells to PTH treatment was determined by RT-qPCR *via* the change in osteogenic marker expression levels characterized above. log_2_-fold change of gene expression in 3D samples on day 14 after 24 h of PTH treatment compared to untreated samples is shown. Data is presented as mean ± SD, *n* = 3. Two-way ANOVA with Šídák's multiple comparisons test was performed comparing the expression levels of cells in fast- and slow-relaxing gels with and without PTH treatment. **p = 0.01 to 0.001.

Functional osteocytes form extensive networks *in vivo* and are connected with each other *via* gap junctions. Using fluorescence recovery after photobleaching (FRAP), it is possible to visualize such connectivity *via* the transfer of fluorescent molecules from a donor cell to the photobleached acceptor cell.^27^ To investigate whether the IDG-SW3 cells embedded in the IPN hydrogels were able to form such functional connections, a FRAP experiment was conducted for cells cultured in the slow-relaxing composition for 14 days. Successful calcein fluorescence recovery could be observed after photobleaching. As the dye is transferred from a donor to the acceptor, the donor cell intensity decreases while the acceptor cell intensity increases and reaches a plateau below the pre-bleaching intensity level (Fig. 6A and B).

By administering the gap junction inhibitor 18α-glycyrrhetinic acid (AGA), a negative control condition with inhibited dye transfer over gap junction could be established (Fig. 6C). This enabled us to confirm gap junctional activity at the observed cell–cell-contacts and exclude major contributions from other potential mechanisms of fluorescent dye uptake. In untreated samples, the connected cells within the slow-relaxing gels were able to regain up to >60% of their initial fluorescent intensity. The large variation within the treatment groups could be attributed to varying numbers of connecting gap junctions between the cells. Seeding cells at a higher density in future experiments could increase the number of successfully connected cells and further allow for a greater sampling number and more in-depth investigation.

Besides cell connectivity, osteocyte functionality also depends on processing and responding to soluble signals. *In vivo*, osteocytes and the coordination of bone homeostasis are regulated by parathyroid hormone (PTH). For studies of osteocyte models *in vitro*, treatment with PTH fragment 1–34 and the appropriate response in the gene expression levels of osteocyte-specific genes has been used to demonstrate osteocyte-like functionality.^5,6,9^ Generally, a decrease in osteocyte-specific markers including Dmp1 was observed, but Pdpn and RANK ligand tend to increase. We evaluated cellular responsiveness to PTH treatment (50 nM) for 24 h in both fast- and slow-relaxing hydrogels after 14 days of 3D culture. Gene expression levels were analyzed in comparison with untreated control samples (Fig. 6D). As expected, Dmp1 expression was downregulated in both fast- and slow-relaxing compositions, however only showed a significant difference to the untreated control samples in slow-relaxing gels. A trend for upregulation of Pdpn could be seen for the slow-relaxing gels that showed strongest osteocyte-like responses on day 14 so far, but not for the fast-relaxing gels.

Finally, to evaluate whether the embedded cells retained their mechanosensing abilities, we performed live-cell 3D Ca^2+^ imaging in slow-relaxing IPN hydrogels on idenTx 3 chips. The Fluo-4 staining method was validated for the 3D setup by administering GSK1016790A as TRPV4 agonist, which induced prolonged Ca^2+^ signals in selected cells. In a second experiment, much shorter calcium spikes indicative of mechanotransduction were occasionally detected when applying a fluid flow through the IPN hydrogel (ESI Fig. S6 and Videos S2, S3†). The interpretation of this preliminary data is however still limited by the missing knowledge of the IPN hydrogel porosity, affecting both molecular diffusion and the effective shear stress on the cells.

## Conclusion

We established two stiffness-matched alginate–collagen IPN hydrogels with different stress relaxation speeds to study the role of matrix stress relaxation on 3D osteocyte morphogenesis. We then characterized 14-days pre-differentiated IDG-SW3 bone cells as a mechanosensitive osteocyte model and embedded them within the developed IPN gels.

Cell morphological analysis showed enhanced cell spreading in the fast-relaxing composition at early time points, but differences in mean cell area decreased over time. At the same time, however, biomolecular and functional osteocyte markers as well as matrix mineralization appeared to increase with slow matrix relaxation speeds at the end timepoint after 14 days. We believe that although faster stress relaxation can accelerate early cell spreading, it may be unable to supply sufficient mechanical support for long-term osteogenic differentiation. Instead, a minimal ECM elasticity may be required for continued cell–matrix interaction and the successful extension of stable dendrites into the extracellular space. It is noteworthy that both our fast- and slow-relaxing compositions exhibit substantial stress relaxation. Thus, future work is needed to extend the investigation to hydrogels with a wider range of stress relaxation speeds.

Our viscoelastic IPN hydrogels prove to be suitable for the investigation of osteogenic cultures *in vitro* and our findings provide further insights into the influence of matrix mechanics on osteocyte morphogenesis. The next goal would be to improve cell networking in order to better model the osteocyte networks found in bone. An avenue to be investigated is the use of more defined, synthetic hydrogels that allow the decoupling of viscous and elastic properties.^28,29^ This would further allow the combined study of multiple matrix properties, such as stiffness and stress relaxation, and potentially reveal synergistic effects of matrix mechanics on osteocyte morphogenesis. Still, additional mechanical cues by compressive or fluid shear stimulation may be needed to mimic the dynamic nature of the bone microenvironment and enable long-term, more *in vivo*-like tissue cultures. In combination with the herein established Ca^2+^ imaging method, this would open new possibilities to study the mechanosensation and cell–cell communication of osteocytes *in vitro*.

## Materials and methods

### Gel casting

#### Buffers

To reduce the risk of calcium phosphate precipitation from the calcium-crosslinker in phosphate-buffered solutions, a NaCl/HEPES buffer (135 mM NaCl, 20 mM HEPES, pH 7.4) was used during the casting process.^30^ After gelation, a NaCl/HEPES/CaCl_2_ buffer supplemented with 3 mM CaCl_2_ was necessary to stabilize the gels during all subsequent washing and incubation steps.

#### Stock solutions

Alginate stock solutions were prepared in 150 mM NaCl and completely dissolved on a shaker at 4 °C for 48 h. For material characterization, PRONOVA UP VLVG sodium alginate (NovaMatrix, 4200501) and PRONOVA UP MVG sodium alginate (NovaMatrix, 4200101) were prepared at a stock concentration of 1% (w/v). For cell culture, the sterile, RGD-functionalized sodium alginates NOVATACH VLVG-4GRGDSP (NovaMatrix, 4270519) and NOVATACH MVG-GRGDSP (NovaMatrix, 4270129) were dissolved at 4% (w/v) and 3% (w/v), respectively. High concentration collagen I from rat tail (8.91 mg mL^−1^, Corning, 354249) was used directly.

#### Hydrogel precursor solutions

All precursor solutions were prepared on ice immediately before gel casting. Alginate stock solutions were diluted to 0.75% (w/v) while the collagen I gel precursor was prepared according to the manufacturer's protocol to a target concentration of 0.75% (w/v). In short, the collagen I stock solution volume *C* was determined based on the total required precursor solution volume *T*. The volume of 10× NaCl/HEPES buffer containing 0.159 g L^−1^ phenol red was 0.1 × *T*, and the volume of 1 M NaOH was estimated as 0.023 × *C*. Milli-Q water was added to reach the total volume *T* and the phenol red indicator color was used to adjust to pH 7.4. Still working on ice, the final mixed solutions for mechanical tests were assembled from the precursor solutions with an alginate : collagen : NaCl/HEPES volume ratio of 2 : 2 : 1, where the third component NaCl/HEPES buffer replaces the cell suspension added to cellular gels. This resulted in final concentrations of 0.3% (w/v) alginate and 0.3% (w/v) collagen I.

#### Gel casting

To cast the alginate–collagen hydrogels, a protocol was adapted from Gillette *et al.*^17^ Key steps are depicted in ESI Fig. S7.† Molds were prepared with a 1 mm thick PDMS sheet (McMaster-Carr, 3788T32) and biopsy punches with 5 mm or 8 mm diameter depending on the experimental requirements. Molds were then assembled on coverslips and placed in well plates for casting. A volume of 25 μL and 60 μL of the final solution was loaded in 5 mm and 8 mm wells respectively. This results in a slight over-filling and prevents air entrapment. Each mold was then covered with a cellulose filter paper pre-wetted with Milli-Q water to flatten and humidify the gel. Then, the collagen was crosslinked at 37 °C for 20 minutes, before the molds were covered with the corresponding CaCl_2_ crosslinking solution and incubated at 37 °C for another 30 minutes to allow the Ca^2+^ ions to diffuse into the gel and crosslink the alginate polymers (Fig. 1B). Fast-relaxing VLVG-containing gels were crosslinked with 20 mM CaCl_2_ and slow-relaxing MVG-containing gels with 5 mM CaCl_2_, to achieve stiffness-matching properties. The gels were finally recovered by dissociating the PDMS mold from the coverslip using tweezers and spatulas. Prior to mechanical testing, gels were swelled for 24–48 h in storage solution (150 mM NaCl, 3 mM CaCl_2_) at 4 °C.

### Mechanical characterization

Non-RGD modified alginates were used for mechanical characterization under the assumption that the mechanical properties would not greatly be affected by the RGD-functionalization required for cell experiments.

#### Unconfined compression testing

The Texture Analyser TA.XTplus (Stable Micro Systems) with a 500 g load cell was used to conduct unconfined compression tests combining stress–strain and stress relaxation measurements in a single protocol.^18,35^ Samples of 8 mm diameter were placed under the parallel plate, which was lowered until in contact with the gel surface (*F*_N_ > 0 N). The gels were compressed at a test speed of 0.02 mm s^−1^ up to a strain of 15%. Then the strain was held constant to record stress relaxation over time for up to 10 minutes. The Young's modulus was calculated by the slope of the stress–strain curve for the range of 5–15% strain. For stress relaxation evaluation, the maximum stress was used as initial value and stress relaxation time was determined as the time needed to reach half of this value.

#### Rheology

Rheological measurements were made on a Modular Compact Rheometer MCR302 (Anton Paar) with an 8 mm parallel plate (PP08), a glass bottom plate, a gap height of 0.75 mm and at 25 °C. The measuring plate was lowered onto the 8 mm diameter gels and surrounded by storage solution (150 mM NaCl, 3 mM CaCl_2_) to prevent gel desiccation. Samples were allowed to equilibrate for 1 minute before measurements and recover for 5 minutes between measurements.

Amplitude sweeps were carried out at 1 Hz oscillating shear strain of 0.01–6.85%. Storage moduli were calculated from the average value in the range of 0.101–0.685% strain, which was linear for all gels (ESI Fig. S1†). After 5 minutes of recovery, stress relaxation tests were performed by preconditioning the samples at a 1 Hz oscillating strain of 0.5% for 3 minutes, then suddenly increasing the strain to a constant 10% while monitoring the shear stress.^31^ Measurement time points were logarithmically ramped from 0.01 s to increase the resolution of the initial relaxation. The thereby generated noise was removed using a moving median filter with a window size of 30 points, followed by a low-pass filter with a constant cut-off frequency of 0.5 on all data points after 10% strain is reached. Then, the measured shear stress was normalized to the initial value defined as the first value at 10% shear strain. The relaxation half-time was calculated based on the time needed to reach half of the initial shear stress.

### 2D cell culture and pre-differentiation

IDG-SW3 bone cells (Kerafast Inc.) were cultured with a protocol adapted from the provider's instructions. To support cell adhesion in 2D culture, tissue culture flasks were coated with collagen I (Sigma-Aldrich, C3867). Cells were seeded at a density of 3000 cells per cm^2^ under immortalizing conditions in expansion medium (alpha MEM (Sigma-Aldrich, M0644) with 10% fetal bovine serum (FBS, Gibco, 10270-106), 50 U mL^−1^ recombinant mouse interferon-gamma (IFN-γ, Gibco, PMC4031) and 1% antibiotic–antimycotic (Gibco, 15240062)) and cultured at 33 °C and 5% CO_2_. At 90% confluence, usually after 2 days, cells were detached using 0.05% trypsin for splitting. To induce osteocytic cell differentiation, the expansion medium was replaced by differentiation medium (alpha MEM, 10% FBS, 50 μg mL^−1^ ascorbic acid (Sigma-Aldrich, A8960), 4 mM β-glycerophosphate (β-GP, Acros Organics, 41099) and 1% antibiotic–antimycotic) at 80% confluence for cell passages between 13 and 17, and incubation was continued at 37 °C and 5% CO_2_. Medium was exchanged twice per week and cultures were washed with phosphate buffered saline (PBS) in between. To dissociate the differentiating cells on day 14, 0.05% trypsin was supplemented with 1.5 U μL^−1^ collagenase type II (Gibco, 17101015) to liberate the cells from their secreted collagen matrix. The washed cell suspension was passed through a cell strainer to remove matrix bits and resuspended in alpha MEM.

### 2D live-cell calcium imaging

For 2D Ca^2+^ imaging, μ-slides VI^0.4^ (ibidi, 80606) were used and collagen coated as described above for tissue culture flasks. IDG-SW3 cells were pre-differentiated for 12 days and reseeded in the μ-Slide channels (*w* = 0.38 mm, *h* = 0.04 mm) at a density of 25 000 cells per cm^2^. They were left to attach during an incubation period of 1.5 h at 37 °C and 5% CO_2_, before the reservoirs were filled with differentiation medium, and incubation was continued. Medium was exchanged daily.

The calcium dye staining solution was prepared as per the manufacturer's recommendations, mixing the 1 mM Fluo-4 AM stock (Invitrogen, F14201) 1 : 1 with 20% Pluronic® F-127 (Sigma-Aldrich, P2443) in DMSO and diluting this 1 : 100 in phenol-red-free alpha MEM to obtain a final concentration of 5 μM Fluo-4 AM and 0.1% Pluronic® F-127. On day 14 of pre-differentiation, cells were washed once with warm PBS, stained for 30 minutes at 37 °C, and washed once with Hank's balanced salt solution containing Ca^2+^ (HBSS, Gibco, 14025050). Channel openings were capped with pieces of autoclaved Microseal sealing film until imaging.

The same HBSS buffer was used for perfusion of the chips. Therefore, 50 mL syringes were filled with pre-warmed HBSS under sterile conditions. At the Leica SP8 confocal laser scanning microscope (CLSM), the syringe, chip and an outlet container were connected to an AL-1000 syringe pump (World Precision Instruments), and the chip was left to rest for 10 minutes, allowing the cells to recover from any disturbing mechanical stimuli. For live-cell imaging, 12 μm *xyzt*-stacks were recorded every 3 seconds for 5 minutes. After 1 minute of static baseline condition, fluid flow was initiated at a flow rate *Q* of 12.93 mL min^−1^. With a buffer viscosity *μ* of 9.4 × 10^−4^ Pa s, this flow rate caused a shear stress *τ* of 2 Pa on the cells according to eqn (1), which can be used to calculate the wall shear stress in a parallel-plate flow chamber.^32^1
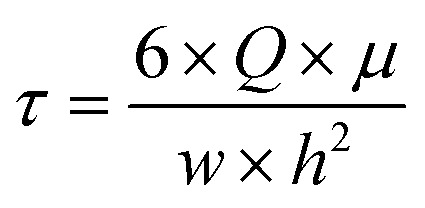


The time series maximum intensity projections (MIPs) were analyzed in ImageJ using the MultiMeasure plugin. Regions of interest (ROIs) were manually drawn around each cell and the intensity values exported. Since IDG-SW3 express Dmp1-GFP at this stage of differentiation, the baseline intensity was defined as the mean intensity during static conditions minus the baseline intensity measured for non-fluorescent human mesenchymal stem cells (Lonza) stained using the same protocol. This baseline was subtracted from the intensity measurements and Ca^2+^ spikes were defined as intensity values exceeding the 9-fold standard deviation of the measurements during the static first minute. This threshold was chosen 3-fold higher than by Degala *et al.*^33^ due to higher noise fluctuations. Finally, the total number of spikes per cell was computed.

### 3D cell embedding, cell culture and viability

To maintain sterile conditions, molds were pre-assembled and autoclaved while the filter papers were cut and sterilized by UV irradiation from both sides. For a final density of 4.5 × 10^6^ cells per mL, pre-differentiated IDG-SW3 were suspended at a 5-fold concentration of 22.5 × 10^6^ cells per mL and held on ice. The alginate and collagen I precursor solutions were prepared under sterile conditions analogously to the description for acellular gel casting and mixed at an alginate : collagen : cell suspension ratio of 2 : 2 : 1. Gels were casted as described above and visualized in Fig. 1E. Gels used for RT-qPCR, PTH treatment and 3D ALP assays were cast as 8 mm discs containing 225 000 cells each, while gels for live/dead quantification, actin-nuclei staining, OsteoImage staining and FRAP were cast with 5 mm diameter containing 90 000 cells.

After successful extraction of the gels from their molds, they were transferred into 24-well plates containing differentiation medium supplemented with 1 mM additional CaCl_2_, resulting in a final concentration of 3 mM. Culture at 37 °C and 5% CO_2_ was continued, and medium was exchanged twice per week.

#### 3D cell culture viability

On days 1 and 7, a live/dead assay was performed using calcein AM (CaAM, Sigma-Aldrich, 56496) as a marker for live cells and ethidium homodimer 1 (EthD1, Sigma-Aldrich, 46043) for dead cell nuclei. In 8-well μ-slides (ibidi), two replicate gels per condition were washed with NaCl/HEPES/CaCl_2_ buffer and incubated in a staining solution with a CaAM (1 : 500) and EthD1 (1 : 1000) for 15 minutes at 37 °C. Subsequently, the samples were washed twice, the second time incubating for 10 minutes at 37 °C, and immersed in phenol red-free alpha MEM for imaging. At a Leica SP8 CLSM, three 100 μm *z*-stacks were taken per replicate. An automated analysis was conducted in ImageJ, analyzing particles in MIPs with live particle sizes >50 μm^2^ and dead particle sizes limited to a range of 10–200 μm^2^. The percentage of viable cells was calculated as the fraction of live cells over the sum of live and dead cells.

### 3D morphological analysis

At 6 h, 24 h (d1), 48 h (d2) and 7 days after cell embedding, three replicate gels per condition were washed three times with warm NaCl/HEPES/CaCl_2_, fixed in 4% paraformaldehyde (PFA, Thermo Scientific, 28908) in NaCl/HEPES/CaCl_2_ for 20 minutes at 37 °C and washed again three times with NaCl/HEPES/CaCl_2_. All solutions for actin-nuclei staining were prepared in NaCl/HEPES/CaCl_2_ buffer. At room temperature (RT), the samples were first blocked in 1% bovine serum albumin (BSA, Sigma-Aldrich, A4503) for 1 h, permeabilized with 0.2% Triton X-100 (Sigma-Aldrich, 93426) for 10 minutes and washed three times with NaCl/HEPES/CaCl_2_. A staining solution with a 1 : 1000 dilution of Hoechst 33342 and 1 : 1000 dilution of phalloidin CruzFluor™ 647 (Santa Cruz Biotechnology, sc-363797) was applied for 1 h in the dark, then samples were washed three times in NaCl/HEPES/CaCl_2_.

At the Leica SP8 CLSM, the gels were imaged at two to three positions capturing 36 μm *z*-stacks. Automated cell area analysis was conducted in ImageJ. MIPs of both channels were segmented with a manually set, fixed threshold and the mean cell area was computed by dividing the area of actin particles by the number of nuclei. Dendrite quantification was performed using the IMARIS automated dendrite tracking software with a dendrite diameter set to 0.8 μm.

### OsteoImage mineral staining

#### Fixation and cryosectioning

For the quantification of mineral deposition within the gels, duplicate 5 mm gels per condition were collected on day 1 and day 14. The hydrogels were washed three times with warm NaCl/HEPES/CaCl_2_, fixed in 4% PFA in NaCl/HEPES/CaCl_2_ for 20 minutes at 37 °C and washed again three times with NaCl/HEPES/CaCl_2_. For cryoprotection, the samples were first incubated with 30% (w/v) sucrose in NaCl/HEPES/CaCl_2_ at 4 °C over night, then in a 1 : 1 mix of 30% sucrose and Tissue-Tek O.C.T. Compound (Sakura Finetek, 4583) for 4 h at 4 °C. Finally, the gels were transferred into cryomolds, covered in O.C.T. Compound and frozen on a cooling block immersed in liquid nitrogen. The samples were cryosectioned on a CryoStar NX70 Cryostat (Thermo Scientific) with both sample temperature and blade temperature at −20 °C. Sections of 20 μm thickness were transferred directly from the blade holder to a Superfrost glass slide. Slides were shortly kept at room temperature before collecting a second section on the same slide and kept frozen at −20 °C until staining.

#### OsteoImage staining

The OsteoImage Mineralization Assay (Lonza, PA-1503) fluorescent reagent was used to specifically stain hydroxyapatite present in the samples. Samples were thawed, washed by dipping the slides in NaCl/HEPES/CaCl_2_ for 5 minutes and stained in duplicates with a solution of OsteoImage reagent (1 : 100) and Hoechst 33342 (1 : 200) in 0.1% TritonX-100 in NaCl/HEPES/CaCl_2_. Additionally, two day 14 sections per condition were stained only with Hoechst as a control. After 30 minutes incubation in the dark, samples were washed three times for 5 minutes. Finally, each section was mounted with a drop of Mowiol and a #1.5 coverslip, which was sealed with nail polish after having dried.

At the Leica SP8 CLSM, 60 μm *z*-stacks spanning the entire sample were acquired at two positions per section. Automated particle analysis in ImageJ was used to quantify the fluorescent signal. MIPs of both channels were segmented with a manually set, fixed threshold and the mean OsteoImage-stained area per cell was computed by dividing the total area of OsteoImage signal by the number of nuclei.

### Cell connectivity analysis by FRAP

To verify the cell–cell connectivity in 3D culture, fluorescence recovery after photobleaching (FRAP) was employed, visualizing the transfer of fluorescent dye molecules over gap junctions between functionally connected donor and acceptor cells. FRAP was performed on day 14 of 3D culture following an adapted protocol from Kuzma-Kuzniarska *et al.*^34^ Cells were stained with CaAM, which is converted to fluorescent calcein intracellularly and can be transferred over gap junctions. For the gap junction inhibition treatment, a 100 mM 18α-glycyrrhetinic acid (AGA) stock solution was prepared in sterile DMSO, all other solutions were prepared in NaCl/HEPES/CaCl_2_ buffer. The 5 mm gels were washed once with warm buffer, transferred into CaCl_2_-supplemented differentiation medium with 100 μM AGA or DMSO as vehicle control and incubated at 37 °C for 90 minutes. Afterwards they were washed again, incubated in a staining solution of CaAM (1 : 1000) for 20 minutes at 37 °C, washed twice, immersed in phenol-red-free alpha MEM again containing 100 μM AGA or vehicle control.

At the Leica SP8 CLSM, FRAP was performed using the FRAP module of the LAS X microscope software. A ROI was manually selected around the target cell for bleaching with 100% 488 nm laser power at the center height of the *z*-stack. Pre- and post-bleaching, 30 μm *z*-stacks with 5 slices were excited with the same 488 nm laser at 0.5% power. Two pre-bleach stacks were acquired with a 10 seconds interval, then the target cell was bleached using 2 iterations and finally post-bleach stacks were acquired for up to 7.5 minutes every 3 seconds. FRAP was performed in slow-relaxing gels for control cells and AGA-treated cells.

In ImageJ, MIPs were computed, and circular ROIs were drawn around the bleached cell, a background area and a reference cell that was not in contact with the bleached cell. Mean intensity values of the ROIs over time were extracted using the MultiMeasure plugin. The intensity values of the target cells were corrected for the background fluorescence and normalized to the reference cell in order to account for photobleaching during image acquisition. Then the fluorescence recovery after bleaching was computed normalized to the average intensity of the two pre-bleach stacks as 1 and the first stack after bleaching as 0.

### Statistical analysis

Calculations and linear regressions were performed either in Excel or Python. Statistical analyses were all performed using GraphPad Prism 9.1.0. Welch's *t* tests were used for pairwise comparison. Welch's one-way ANOVA with Dunnett's T3 multiple comparisons test and two-way ANOVA with Šídák's multiple comparisons test were used for comparisons between more than two experimental groups. When normality could not be assumed, non-parametric Mann Whitney test and Kruskal–Wallis test followed by Dunn's multiple comparisons test were used for two or more groups respectively. Significances are indicated by the *p*-value ranges *p = 0.05 to 0.01, **p = 0.01 to 0.001, ***p = 0.001 to 0.0001, ****p ≤ 0.0001.

## Author contributions

X.-H. Q. and M. B. conceptualized the research. M. B. and D. Z. performed the experiments. M. B. and X.-H. Q. analyzed the data. X.-H. Q. and R. M. supervised the project. M. B. prepared the first draft of the manuscript. All authors discussed the results and reviewed the manuscript.

## Conflicts of interest

There are no conflicts to declare.

## Supplementary Material

BM-012-D3BM01781H-s001

BM-012-D3BM01781H-s002

BM-012-D3BM01781H-s003

BM-012-D3BM01781H-s004

## References

[cit1] Jacobs C. R., Temiyasathit S., Castillo A. B. (2010). Annu. Rev. Biomed. Eng..

[cit2] Kato Y., Windle J. J., Koop B. A., Mundy G. R., Bonewald L. F. (1997). J. Bone Miner. Res..

[cit3] Kato Y., Boskey K., Spevak L., Dallas M., Hori M., Bonewald L. F. (2001). J. Bone Miner. Res..

[cit4] Spatz J. M., Wein M. N., Gooi J. H., Qu Y. L., Garr J. L., Liu S., Barry K. J., Uda Y., Lai F., Dedic C., Balcells-Camps M., Kronenberg H. M., Babij P., Pajevic P. D. (2015). J. Biol. Chem..

[cit5] Wang K., Le L. S., Chun B. M., Tiede-Lewis L. M., Shiflett L. A., Prideaux M., Campos R. S., Veno P. A., Xie Y. X., Dusevich V., Bonewald L. F., Dallas S. L. (2019). J. Bone Miner. Res..

[cit6] Woo S. M., Rosser J., Dusevich V., Kalajzic I., Bonewald L. F. (2011). J. Bone Miner. Res..

[cit7] Aziz A. H., Wilmoth R. L., Ferguson V. L., Bryant S. J. (2020). ACS Appl. Bio Mater..

[cit8] Wilmoth R. L., Ferguson V. L., Bryant S. J. (2020). Adv. Healthc. Mater..

[cit9] Yang Y., Wang M., Yang S., Lin Y., Zhou Q., Li H., Tang T. (2020). Biofabrication.

[cit10] Chaudhuri O., Gu L., Klumpers D., Darnell M., Bencherif S. A., Weaver J. C., Huebsch N., Lee H. P., Lippens E., Duda G. N., Mooney D. J. (2016). Nat. Mater..

[cit11] McGarrigle M. J., Mullen C. A., Haugh M. G., Voisin M. C., McNamara L. M. (2016). Eur. Cells Mater..

[cit12] Bessot A., Gunter J., Waugh D., Clements J. A., Hutmacher D. W., McGovern J., Bock N. (2023). Adv. Healthc. Mater..

[cit13] Hadjab I., Farlay D., Crozier P., Douillard T., Boivin G., Chevalier J., Meille S., Follet H. (2021). J. Biomech..

[cit14] Lee K. Y., Mooney D. J. (2012). Prog. Polym. Sci..

[cit15] Zhao X., Huebsch N., Mooney D. J., Suo Z. (2010). J. Appl. Phys..

[cit16] Charbonier F., Indana D., Chaudhuri O. (2021). Current Protocols.

[cit17] Gillette B. M., Jensen J. A., Wang M., Tchao J., Sia S. K. (2010). Adv. Mater..

[cit18] Darnell M., Young S., Gu L., Shah N., Lippens E., Weaver J., Duda G., Mooney D. (2017). Adv. Healthc. Mater..

[cit19] Nam S., Hu K. H., Butte M. J., Chaudhuri O. (2016). Proc. Natl. Acad. Sci. U. S. A..

[cit20] Moharrer Y., Boerckel J. D. (2021). Bone.

[cit21] Klein-Nulend J., Bakker A. D., Bacabac R. G., Vatsa A., Weinbaum S. (2013). Bone.

[cit22] Lu X. L., Huo B., Park M., Guo X. E. (2012). Bone.

[cit23] Bonewald L. F. (2011). J. Bone Miner. Res..

[cit24] Schädli G. N., Vetsch J. R., Baumann R. P., de Leeuw A. M., Wehrle E., Rubert M., Müller R. (2021). Commun. Biol..

[cit25] Seo J., Shin J. Y., Leijten J., Jeon O., Ozturk A. B., Rouwkema J., Li Y. C., Shin S. R., Hajiali H., Alsberg E., Khademhosseini A. (2018). ACS Appl. Mater. Interfaces.

[cit26] Loebel C., Mauck R. L., Burdick J. A. (2019). Nat. Mater..

[cit27] Abbaci M., Barberi-Heyob M., Stines J. R., Blondel W., Dumas D., Guillemin F., Didelon J. (2007). Biotechnol. J..

[cit28] Tang S. C., Richardson B. M., Anseth K. S. (2021). Prog. Mater. Sci..

[cit29] Zhang K. Y., Feng Q., Fang Z. W., Gu L., Bian L. M. (2021). Chem. Rev..

[cit30] Palazzolo G., Broguiere N., Cenciarelli O., Dermutz H., Zenobi-Wong M. (2015). Tissue Eng., Part A.

[cit31] Valentin J. D. P., Qin X.-H., Fessele C., Straub H., van der Mei H. C., Buhmann M. T., Maniura-Weber K., Ren Q. (2019). J. Colloid Interface Sci..

[cit32] Wittkowske C., Reilly G. C., Lacroix D., Perrault C. M. (2016). Front. Bioeng. Biotechnol..

[cit33] Degala S., Zipfel W. R., Bonassar L. J. (2011). Arch. Biochem. Biophys..

[cit34] Kuzma-Kuzniarska M., Yapp C., Pearson-Jones T. W., Jones A. K., Hulley P. A. (2014). J. Biomed. Opt..

[cit35] Qiu W., Gehlen J., Bernero M., Gehre C., Schädli G. N., Müller R., Qin X.-H. (2023). Adv. Funct. Mater..

