# Supplementary material for: Interpenetrating network hydrogels for studying the role of matrix viscoelasticity in 3D osteocyte morphogenesis
Source: Biomater Sci. 2024 Jan 10;12(4):919–32. doi: 10.1039/d3bm01781h (PMC10863643; doi:10.1039/d3bm01781h)
Supplement: BM-012-D3BM01781H-s001 [file BM-012-D3BM01781H-s001.pdf]

Electronic Supplementary Material (ESI) for Biomaterials Science.
This journal is © The Royal Society of Chemistry 2024

Biomater. Sci., 2024, DOI: 10.1039/D3BM01781H

Supporting Information

Interpenetrating Network Hydrogels for Studying the Role of Matrix Viscoelasticity in 3D Osteocyte Morphogenesis

Margherita Bernero,[a] Doris Zauchner, [a] Ralph Müller[a] and Xiao-Hua Qin*[a]

[a]Institute for Biomechanics, ETH Zürich, Switzerland.

E-mail: qinx@ethz.ch

This PDF file includes:

Supplementary Methods

Supplementary Figures S1 to S7

Supplementary References

Other supplementary materials include:

Supplementary Videos S1 to S3

Supplementary Methods

Dmp1-GFP reporter tracking

To follow the osteoblast-to-osteocyte transition during culture under osteogenic conditions using the Dmp1-GFP marker, expansion cells were seeded at a density of 3500 cells per cm^2 onto collagen coated 20 mm glass coverslips in a 12-well plate. On days 1, 7, 14 and 28 in differentiation medium, three replicate wells were fixed with 4% paraformaldehyde (PFA, Thermo Scientific, 28908) for 10 minutes at 37°C. At room temperature (RT), the samples were first blocked with 1% bovine serum albumin (BSA, Sigma-Aldrich, A4503) in PBS for 1 h, permeabilized with 0.2% Triton X-100 (Sigma-Aldrich, 93426) in PBS for 10 minutes, washed three times with PBS and finally, nuclei were stained with Hoechst 33342 at a dilution of 1:1000 in 1% BSA for 1 h. The samples were washed three times with PBS and then imaged on a Leica SP8 confocal laser scanning microscope (CLSM). In the microscope, the sample coverslips were mounted on a second, 30 mm glass coverslip and moisturized with a few drops of PBS. Per replicate, three 100 µm *z*-stacks were taken.

A CellProfiler pipeline was implemented to quantify the fraction of GFP-positive cells. Nuclei were first identified by Ostu thresholding and segmentation of GFP-positive cells was performed with a manually set threshold. Cells were then classified as GFP-positive via the co-localization of GFP signal with each nucleus. The mean GFP-intensity of GFP-positive cells was calculated per nucleus area and cells with a mean intensity below 5×10^{-7} were filtered out.

Alkaline phosphatase (ALP) assay and DNA assay

ALP and DNA sample preparation. A colorimetric alkaline phosphatase (ALP) assay kit (abcam, ab83369) was employed to test for the ALP activity as a marker for osteoblastic differentiation both in 2D differentiating cells and in 3D culture over the course of 28 days in total. As baseline measurement, 3x 500'000 expansion cells (day 0) were harvested during splitting, washed, and resuspended in 250 µL ALP assay buffer. For 2D differentiation, 500'000 expansion cells were seeded per well of a collagen coated 6-well plate and cultured under differentiation-inducing conditions. On day 7 and 14, cell samples were collected from three replicate wells each by trypsinization, washed and finally resuspended in 250 µL ALP assay buffer. For the assay of 3D culture samples, three replicate gels of 8 mm diameter were used per condition and collected on days 1, 7 and 14. Gels were washed once with NaCl/HEPES/$CaCl_2$ and transferred into 250 µL ALP assay buffer. Immediately after collection, all samples were homogenized using autoclaved pellet pestles and the pestle motor, flash frozen in liquid nitrogen and stored at -80 °C until use.

ALP assay. The ALP assay was performed according to the manufacturer's instructions. Specifically, the samples were thawed on ice to prevent protein degradation, centrifuged at maximum speed at 4°C for 15 minutes to remove any insoluble material and the supernatant collected in a new tube. The assay setup was planned for technical triplicates of each sample, duplicates of the standard dilutions and three background controls per 3D condition. Sample wells were loaded with 50 µL sample supernatant while background wells were loaded with 10 µL sample supernatant and 20 µL stop solution. The appropriate amounts of 5 mM *p*-nitrophenyl phosphate (pNPP) substrate solution and ALP assay buffer were added to all wells and the standard curve was prepared as instructed. The plates were incubated at RT in the dark for 1 h, then the reactions were stopped using 20 µL stop solution. Plates were shaken gently and absorption at 405 nm was measured on a Spark 10M plate reader (Tecan). The remaining sample supernatants were frozen at -20 °C to be used for DNA quantification.

To evaluate the ALP assay data, measurements were corrected by the blank as well as the background control (for 3D samples only). The technical replicates were averaged and quantified based on the standard curve. Finally, ALP activity was normalized by the DNA content determined in the DNA quantification assay as described next.

DNA assay. Frozen samples from the ALP assay were thawed and incubated at RT for 48 h. Then, DNA quantification was performed using the Quant-iT PicoGreen dsDNA assay kit (Invitrogen, P7589) following the manufacturer's protocol and the plate setup was planned analogously to the ALP assay. In short, the standard dilution was prepared as instructed and loaded into a 96-well plate. Sample and background wells were loaded with 12.5 µL sample supernatant as well as the appropriate amount of TE buffer. 100 µL PicoGreen solution was added to sample and standard wells. The plate was shaken and incubated for 5 minutes in the dark. Finally, after shaking again, the emission at 535 nm was measured with an excitation at 485 nm on a plate reader.
For DNA content quantification, the blank was subtracted from all measurements and the background control from each sample measurement. The technical replicates were averaged, quantified based on the standard curve and the initial amount of DNA per sample was computed.

Parathyroid hormone (PTH) treatment
PTH treatment was performed following the protocol described by Yang *et al*.[1] During media exchange on day 14 of 3D culture, new differentiation medium supplemented with either 50 nM PTH (bovine fragment 1-34, Sigma-Aldrich, P3671) or PBS as vehicle control was added. Culture at 37 °C and 5% CO_2 was continued for 24 h until RNA was isolated as described below.

Gene expression by real-time quantitative PCR (RT-qPCR)
RT-qPCR sample preparation. Osteogenic marker gene expression was quantified on 2D pre-differentiating cells, 3D embedded cells and to validate cell response to PTH treatment. Three times 0.5×10^6 cells were collected from 2D cultures of expansion cells and after 14 days of pre-differentiation, and stored in RNAlater solution (Invitrogen, AM7020) at 4 °C. On days 1, 7 and 14 (control and after PTH treatment), six 8 mm hydrogels per condition were washed and stored in RNAlater at 4 °C. To obtain a sufficient amount of mRNA, two 8 mm hydrogels were combined for one analysis, resulting in three samples per condition.

RNA isolation. Once all samples had been collected, total RNA was isolated. RNAlater solution was removed by dilution and pelleting of cells in suspension, or by aspiration in the case of 3D hydrogels. For sample disruption and homogenization, samples were resuspended in 300 µL TRIzol reagent (Invitrogen, 15596026) and crushed using autoclaved pellet pestles on ice. Another 300 µL TRIzol were added, then samples were centrifuged at 8500 g for 30 seconds at RT and the supernatant was collected. RNA isolation was performed using the RNeasy Micro Kit following the manufacturer's instruction, using QIAShredder columns for additional homogenization and including DNA removal using the RNase-Free DNase kit as per the manufacturer's instructions. RNA was eluted in 30 µL RNase-free water and frozen at -20 °C.

cDNA synthesis by reverse transcription. RNA samples were thawed on ice and the RNA concentration measured by Nanodrop spectrophotometry. For cDNA synthesis, 240 ng of RNA were reverse transcribed in a 20 µL reaction using the PrimeScript RT Master Mix (TaKaRa, RR036A) following the manufacturer's instructions. In short, required RNA eluate volumes were combined with 4 µL PrimeScript RT Master Mix and up to 20 µL RNase-free water. Reverse transcription was performed on a T100 Thermal Cycler (Bio-Rad) using the following protocol: 15 min at 37 °C, 5 s at 85 °C, hold at 4 °C. cDNA samples were then stored at -20 °C.

RT-qPCR. The TaqMan Fast Universal PCR Master Mix (Applied Biosystems, 4352042) and TaqMan gene expression assays for selected osteogenic marker genes were used: Alpl (Applied Biosystems, Mm00475834_m1), Pdpn (Mm00494716_m1) and Dmp1 (Mm01208363_m1) with B2m (Mm00437762_m1) as reference gene. The 96-well PCR plate setup was designed using the gene maximization strategy and reactions were run in duplicate with no-template-controls (NTCs). Working on ice, samples were diluted with nuclease-free water and supermixes were prepared by combining

TaqMan gene expression assays with PCR Master Mix according to the manufacturer's calculations. After sequentially adding samples and supermixes, the plate was sealed using Microseal sealing film and the solutions mixed by pulse centrifugation (3x 10 s). RT-qPCR was performed on a CFX96 Real Time C1000 Touch Thermal Cycler (Bio-Rad) following the manufacturer's protocol: 1) 20 s at 95 °C, 2) 1 s at 95 °C, 3) 20 s at 60 °C, 4) back to step 2 for 44x, 5) hold at 4 °C. The C_q measurements were exported, and relative gene expression was quantified implementing the $2^{-\Delta\Delta Cq}$ method as described by Livak and Schmittgen[2]. Fold changes were calculated relative to the lowest detected measurement and normalized by the reference gene, assuming 100% amplification efficiency.

Alizarin Red S staining

Cryosections with 20 µm thickness were prepared as for OsteoImage staining and stained for 1 minute in a solution of 2 mg mL^{-1} Alizarin Red S (Sigma-Aldrich, A5533). They were then washed in milliQ water, dehydrated in acetone, cleared in xylene and finally mounted with DPX mounting medium (Sigma-Aldrich, 06522). Slides were scanned using the Slide Scanner Panoramic 250 (3DHISTECH).

3D live-cell calcium imaging

For 3D Ca^{2+} imaging, idenTx 3 chips (AIM Biotech, DAX-1) were used for both chemical and mechanical stimulation. IDG-SW3 cells were pre-differentiated for 14 days and embedded on-chip at a final density of 4.5×10^6 cells mL^{-1}. The central gel channels were filled with 10 µL hydrogel solution and collagen was allowed to crosslink for 20 minutes at 37 °C and 5% CO_2. Then, calcium crosslinking solution was added into each media channel for alginate crosslinking during another 30 minutes incubation. Finally, the crosslinking solution was replaced with $CaCl_2$-supplemented differentiation medium and cell culture was resumed with media changes every other day.

TRPV4 agonist. For chemically induced Ca^{2+} signaling on day 7, media channels were washed once with NaCl/HEPES/$CaCl_2$ buffer and samples were stained for 1 h at 37 °C using the same Fluo-4 AM solution as described for 2D Ca^{2+} imaging. Thereafter, gels were washed with HBSS containing Ca^{2+} for 30 minutes at 37 °C. For the activation of intracellular Ca^{2+} signaling, GSK1016790A (Cayman Chemicals, 17289) was used as TRPV4 agonist and prepared at a concentration of 20 µM in HBSS. At the Leica SP8 CLSM, 12 µm *xyzt*-stacks were recorded every 3 seconds for 10 minutes. After 1 minute of static baseline condition, TRPV4 agonist solution was manually added by emptying the reservoirs with a tissue paper while injecting the agonist solution into both reservoirs on one side. A pressure gradient between the two media channels was kept by varying the fill volume in order to facilitate the agonist diffusion into the gel channel.

3D fluid flow. In a second experiment using fluid flow stimulation, the gels cultured on-chip for 7 days were additionally treated with alginate lyase (Sigma-Aldrich, A1603) to increase the gel porosity by enzymatic degradation of alginate. This was necessary to facilitate 3D gel perfusion. A 500 U mL^{-1} alginate lyase stock solution was prepared in milliQ water and added to the Fluo-4 AM staining solution at 1:500. The chips were incubated with this solution for 1 h at 37 °C and 5% CO_2 and subsequently washed with HBSS containing Ca^{2+} for 30 minutes at 37 °C. At the Leica SP8 CLSM, two syringe pumps were connected to both openings of a single media channel, while both openings of the second channel were used as outlets into a waste container. After 1 minute of static baseline, flow was initiated at a rate of 100 µL min^{-1} (50 µL min^{-1} from each inlet) and 12 µm *xyzt*-stacks were recorded every 3 seconds for 5 minutes.

The time series images were processed as for 2D Ca^{2+} imaging and intensity values for plotting were normalized by subtracting the first value at baseline.

Supplementary Figure S1

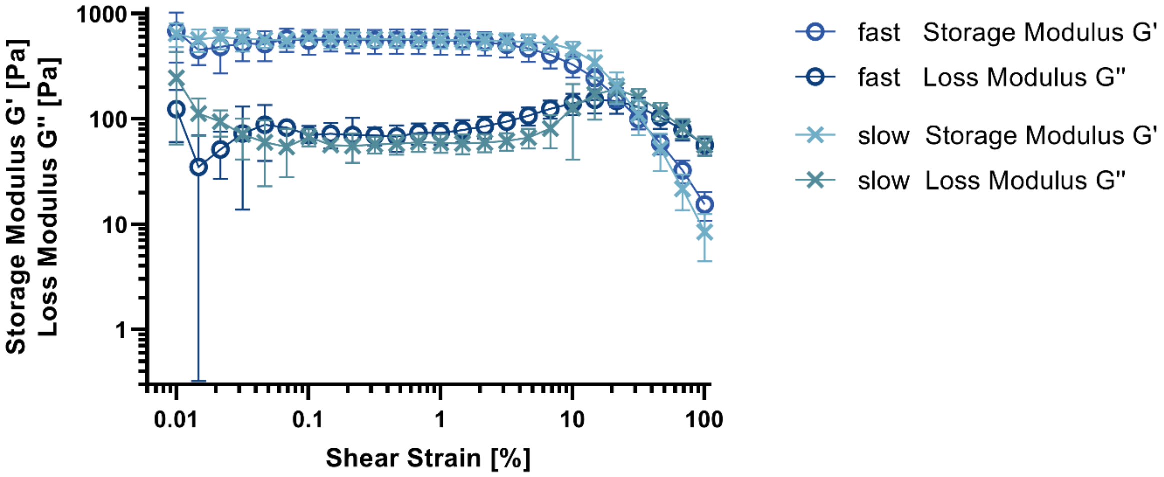

Fig. S1 Rheological amplitude sweeps of swollen IPN hydrogels (diameter: 8 mm) at frequency of 1 Hz and shear strain of 0.01-100% were used to estimate the linear viscoelastic range (LVER). Data points are presented as mean ± SD, *n* = 3.

Supplementary Figure S2

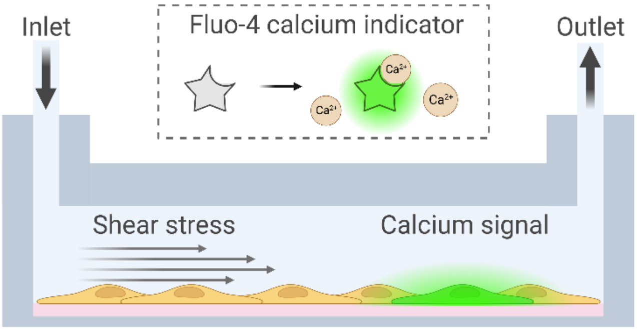

Fig. S2 Schematic of the 2D Ca^{2+} imaging setup. The cells were seeded in ibidi µ-Slides VI $^{0.4}$ and cultured for 2 days to allow attachment and recovery after reseeding. On day 14 of pre-differentiation, cells were stained with Fluo-4 AM, which becomes fluorescent when Ca^{2+} is present and bound to the indicator. By connecting a syringe pump to the channel inlet, fluid flow shear stress can be applied to the cell monolayer. Intracellular Ca^{2+} spikes are visualized by an increase in fluorescent signal which indicates mechanosensation and signal transduction.

Supplementary Figure S3

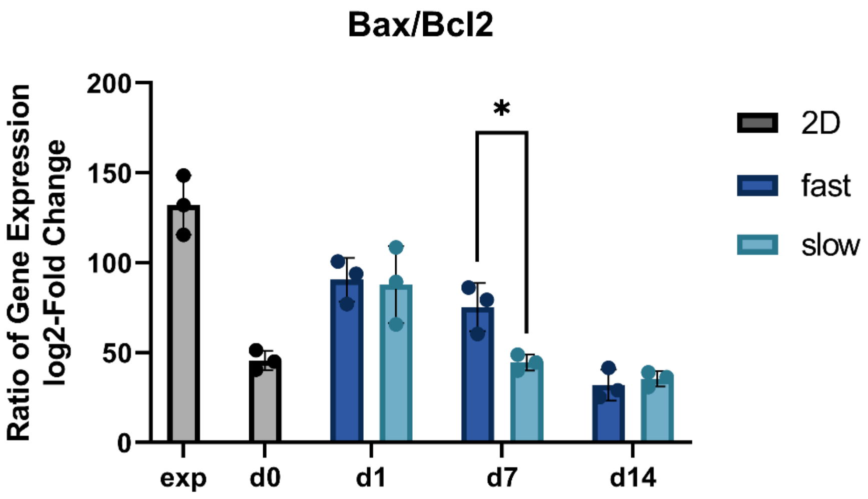

Fig. S3 Apoptotic marker gene expression (Bax: pro-apoptotic, Bcl2: anti-apoptotic, reference gene B2m) was determined by RT-qPCR. The ratio of Bax to Bcl2 expression was used as an indicator for apoptotic stress (higher ratio indicates higher stress). Gene expression was measured for expansion cells, after 14 days of 2D pre-differentiation and on days 1, 7 and 14 of 3D culture. Data is presented as mean ± SD, $n = 3$. Two-way ANOVA with Tukey's multiple comparisons test was performed. * $p = 0.05$ to 0.01.

Supplementary Figure S4

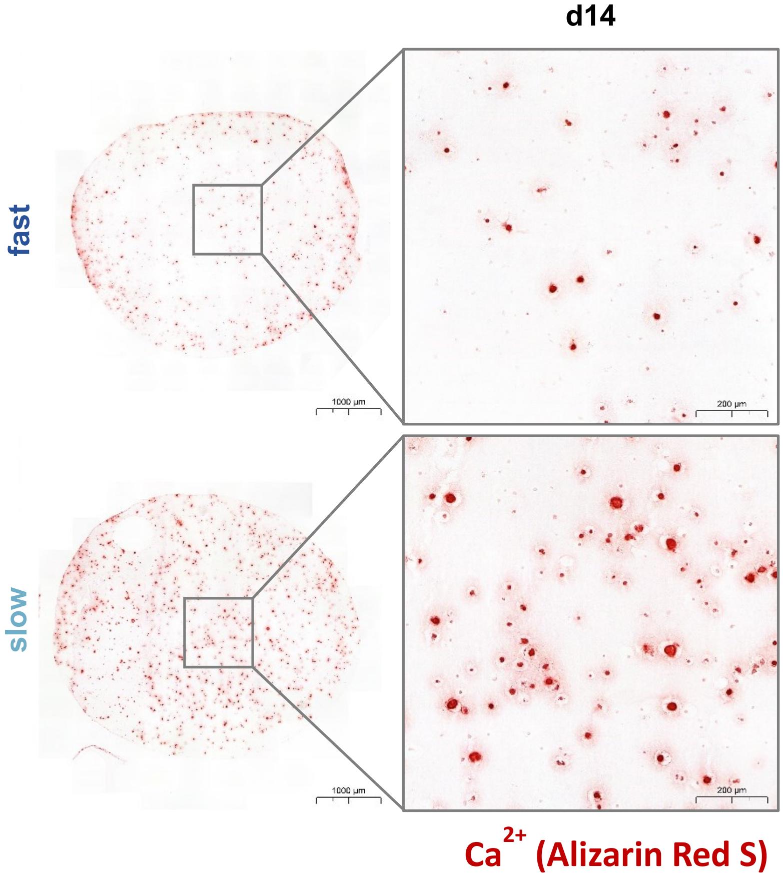

Fig. S4 Matrix mineralization visualized by Alizarin Red S staining for Ca^{2+} in fast- and slow-relaxing IPN hydrogel sections on day 14. Scale bars are 1000 µm (whole gel) and 200 µm (detail).

Supplementary Figure S5

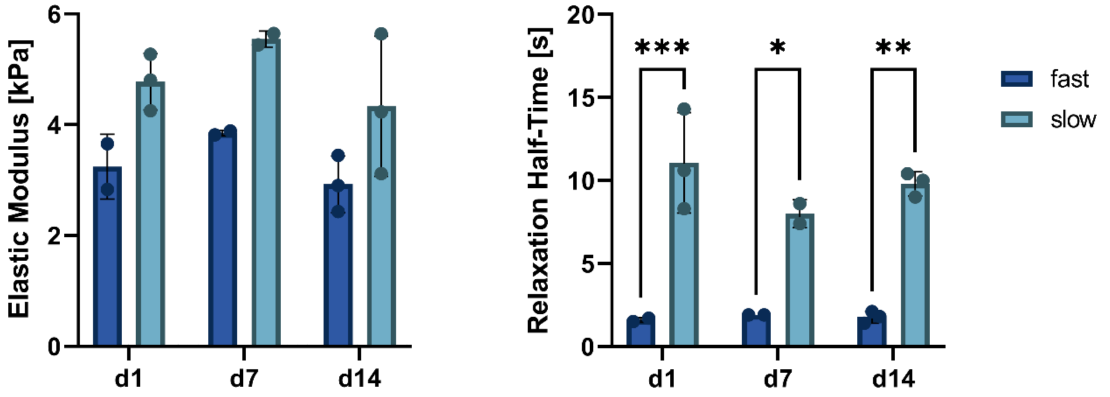

Fig. S5 Mechanical properties of 8 mm hydrogels during 3D cell culture were determined by unconfined compression testing using the Texture Analyser. Visibly damaged gels were excluded from the analysis. The Elastic Modulus was determined from the slope between 5-15% compressive strain while stress relaxation was measured at 15% constant strain. Data is presented as mean ± SD, n = 2-3. Two-way ANOVA with Tukey's multiple comparisons test was performed. * p = 0.05 to 0.01, ** p = 0.01 to 0.001, *** p = 0.001 to 0.0001.

Supplementary Figure S6

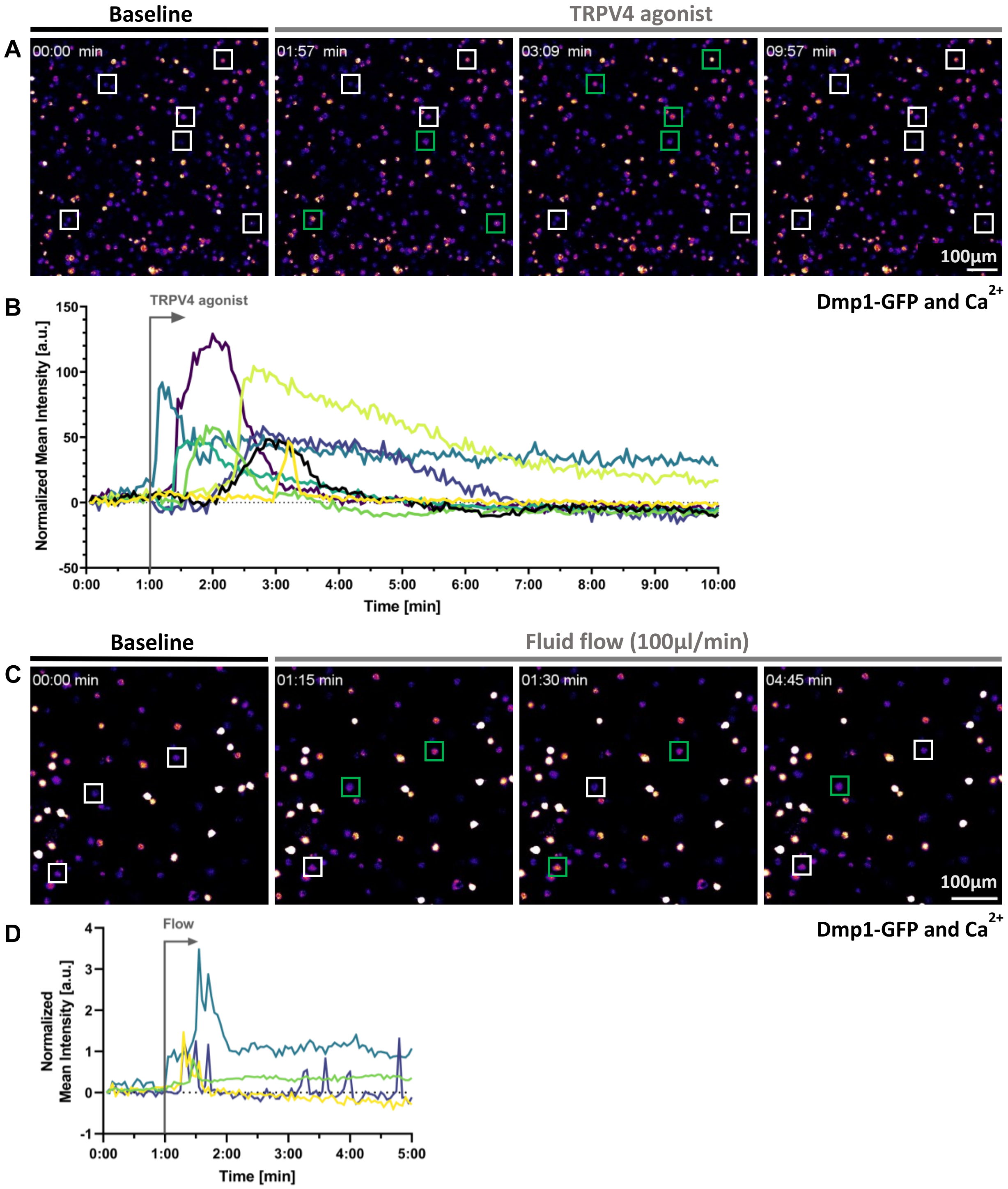

Fig. S6 Proof of concept 3D live-cell Ca^{2+} imaging of pre-differentiated IDG-SW3 cells was performed on day 7 in slow-relaxing gels. Cells were stained with Fluo-4 to emit a Ca^{2+}-concentration dependent fluorescent signal when TRPV4 ion channels were activated by a chemical agonist or when fluid shear stress was applied in idenTx 3 chips. (A and C) Sum slices projections of the cells at baseline and after TRPV4 agonist administration (A) or fluid flow application at 100 µL min^{-1} (C) are shown, visualizing fluorescence intensity (Dmp1-GFP and Ca^{2+} signal overlapping). Selected responsive cells are highlighted and green boxes denote a Ca^{2+} spike. See also **Supplementary Videos S2 and S3**. (B and D) Mean intensity signals of selected responsive cells normalized to the first time point are shown over time. Each line represents an individual cell. The TRPV4 agonist induces intracellular Ca^{2+} spikes of longer duration (B) compared to the mechanically activated cells under fluid shear stress (D). Individual cells show multiple mechanotransduction responses under fluid flow stimulation.

Supplementary Figure S7

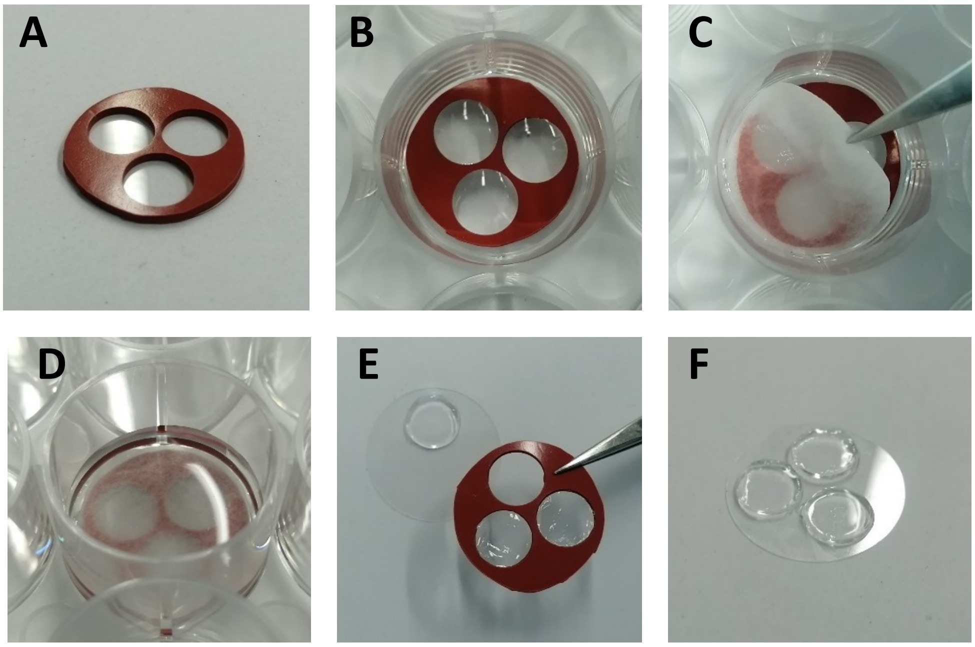

Fig. S7 Gel casting procedure key steps are shown: an assembled mold with 8 mm wells and 20 mm outer diameter (A), wells loaded with the final solution (B), covering with a pre-wetted filter paper (C), alginate crosslinking with a $CaCl_2$ solution (D), the disassembled mold with gels remaining either on the cover slip or within the PDMS mold (E), and the final hydrogels (F). Note that a pure alginate gel is shown here, alginate-collagen IPN gels are more turbid.

References

1. Y. Yang, M. Wang, S. Yang, Y. Lin, Q. Zhou, H. Li and T. Tang, *Biofabrication*, 2020, **12**, 045013.
2. K. J. Livak and T. D. Schmittgen, *Methods*, 2001, **25**, 402-408.
